# Medical Board Organized

**Published:** 1894-07

**Authors:** 


**Medical Board Organized.**—During the recent term of the district court, Judge A. L. McLane appointed Doctors J. P. Arthur, L. M. Berg, J. M. McKnight, W. W. MacGregor, A. W. Wilcox, H. J. Hamilton, T. J. Turpin and F. G. Gongora, of Laredo, and Dr. Layton, of San Diego, as the Board of Medical Examiners for the Forty-ninth Judicial District. They met last Saturday, in Messrs. Arthur, McKnight & Turpin's office, and organized by the election of Dr. J. P. Arthur as President, and Dr. L. M. Berg as Secretary. They will meet again July 25th, for the purpose of examining any candidate who may apply to them for a certificate.

---

**Dr. Swearingen,** the State Health Officer, has issued a circular letter to the county and city physicians of Texas, containing suggestions as to limiting the ravages of consumption by preventive measures,—a timely and sensible paper. A copy may be had upon application. Send for a copy and get your town paper to publish it. The people should be instructed how to avoid the disease.

